# Reliability of Data Collected via Ecological Momentary Assessment on the Example of FeverApp Registry

**DOI:** 10.3390/children10020385

**Published:** 2023-02-15

**Authors:** Larisa Rathjens, Moritz Gwiasda, Silke Schwarz, Ricarda Möhler, David D. Martin, Ekkehart Jenetzky

**Affiliations:** 1 Faculty of Health School of Medicine, Witten/Herdecke University, 58455 Witten, Germany; 2 Department of Pediatrics, Eberhard-Karls University Tübingen, 72076 Tübingen, Germany; 3 Department of Child and Adolescent Psychiatry and Psychotherapy, University Medical Center of the Johannes-Gutenberg-University, 55131 Mainz, Germany

**Keywords:** data quality, reliability, ecological momentary assessment, registry, FeverApp

## Abstract

The FeverApp registry is an ambulant ecological momentary assessment (EMA) model registry focusing on research of fever in children. Verification of EMA reliability is a challenge, due to absence of other source data. To ensure the reliability of EMA data, 973 families were invited to reassess their documentation in a survey. The survey contained questions (a) regarding the number of children, (b) genuineness of entries, (c) completeness of submitted fever episodes, (d) medication, (e) usefulness and further usage of the app. Of those invited, 438 families (45% response rate) participated in the survey. Of these, 363 (83%) families have registered all their children, 208 families have one child. The majority (n = 325, 74.2%) of families stated that they only made genuine entries in the app. Agreement between survey and app for fever episodes is 90% (Cohen’s κ = 0.75 [0.66, 0.82]). Medication shows 73.7% agreement, κ = 0.49 [0.42; 0.54]. The majority (n = 245, 55.9%) consider the app as an additional benefit and 87.3% would like to use it further. Email surveys are a possible approach to evaluate EMA based registry data. The possible observation units (children and fever episodes) show an adequate reliability. With this approach, surveys of further samples and variables could help to improve the quality of EMA based registries.

## 1. Introduction

Quality of data depends on its source. According to Keller et al. [1] data derived from mobile devices are defined as opportunity data associated with lack of transparency and reliability. These are real-time data that are usually unstructured. The designed data from scientific research collected via survey or experiment are depicted as the opposite concerning data quality. The self-reported real-time data captured via electronic diaries in a framework of scientific study are not covered by this partition.

The quality of designed data is often reduced due to recall bias. Ideally, immediate assessment is preferred in context of medical research. Ecological momentary assessment (EMA) is a modern way to report real-time data [2,3,4] in a direct manner without recall bias [5]. Hence, the reliability of data used for scientific research should approach the standards for data quality of clinical data. But EMA relies completely on the compliance of participants in entering data, because usually no additional sources exist.

A possible evaluation of compliance to enter data could be to request answers at a second time, comparable to the concept of retest-reliability. This is then not anymore “momentary” but relies on the “memory” of the participants and depends on motivation. Further, it is an additional effort for the reporting persons. Alternatively, only a reduced set of most important items could be re-evaluated by a second request.

The FeverApp registry [6] has collected data via parental EMA of the child’s febrile episodes since September 2019. The FeverApp as a tool for documentation and information has already been described elsewhere [7]. Recruitment started in a large pediatric reference office. Since July 2020, the FeverApp has been spread in a larger scale to multiple pediatric offices. Until now, pediatric offices solely grant access for parents. The primary goals of the non-profit project are to assess guideline adherence and parental confidence in managing fever, and reduce overuse of antipyretics, antibiotics and health providers [6]. Fever occurs most often in families with young children.

The family is the main observation unit, which consists of caregivers (mother, father, grandmother, grandfather, etc.) and registered children. Each registered child has its own profile. Multiple entries concerning wellbeing, temperature, warning signs and symptoms, doctor visits, diagnosis and delivered medication within a fever episode can be documented in the case of the child’s fever.

Correct handling of childhood fever is essential for the health status in the future: the overuse of antipyretics could cause serious sequelae [8,9,10]. The research of the mechanisms of childhood fever and specifics of fever handling in Germany and other countries could help to improve the health management of future generations. Therefore, the researchers intend to achieve a high quality of the data in the FeverApp registry to conduct valid conclusions in research questions.

Schmidt et al. [11] have underlined that the “context of data quality assessments” is relevant for the evaluation of data quality. For the FeverApp registry it is essential to have a reliable recruitment of the relevant observation units: children and fever episodes. Whereas the amount of participating children could be influenced and can be entered at once, the amount of suddenly occurring fever epsiodes is not influenceable. Nevertheless, the amount of entered fever episodes determs the data analysis. It is desirable for researchers that the users enter reliable information concerning all fever episodes of their children. For instance, reliable information about medication delivery is needed for assessment of the parental adherence concerning medication with antipyretics and antibiotics [6].

The objective of the study is to validate the reliability of the data collected using EMA regarding fever episodes and medication in the FeverApp registry. For this aim, an email-based survey was prepared. The users reported information concerning their FeverApp usage in the past. Another goal of the survey is to evaluate the attitudes of the users toward the app concerning genuineness of entered data, the benefit of the app and the wish to use the app in the future.

## 2. Materials and Methods

### 2.1. FeverApp Registry

The Federal Ministry of Education and Research (BMBF) of Germany has been funding six model registries since 2019 [12]. They should provide features of registries exemplarily, such as consideration of observing (parent using an app) and observed (children) unit at suddenly occurring events (fever episodes) [13]. The registry protocol was published [6] and is registered in the German Clinical Trials Register (DRKS) with registration number DRKS00016591.

The FeverApp registry is designed as an ecological momentary assessment (EMA) of parents on spontaneous capture of a fever event, which is fundamentally different from a physician-managed registry with mandatory fields and regular visits. The collected EMA data rely on event-based sampling at the family level and on time-based sampling at the fever episode level: children have fever occasionally, but the researchers are additionally interested in changes of some parameters over time during a fever episode: e.g., body temperature, parental confidence and children’s wellbeing or amount of medication administrations during a fever episode. The EMA design was combined [14] owing to the multilevel structure of the data.

**Figure 1:**
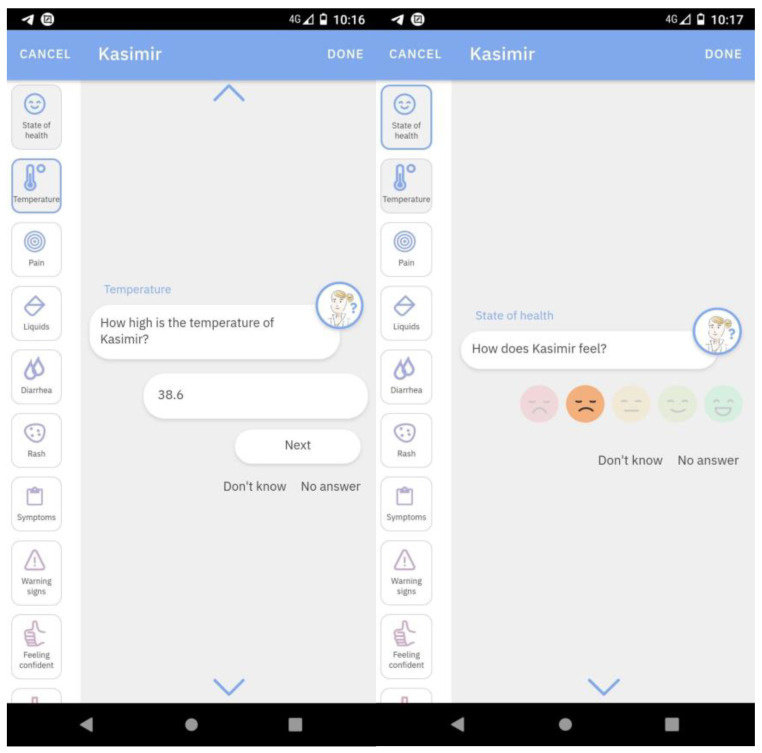
App design sample: Questions about well-being and temperature of a child.

The time to make entries is very variable; it depends on the child’s condition and parental measurements. It could take several minutes. Figure 1 depicts the info library and questions concerning wellbeing of a child, parental confidence and the summary of a fever episode.

### 2.2. Data Protection

In general, the FeverApp could be used completely anonymously, if no identifying entries were made. Currently no mandatory fields force identification. The app is mainly distributed by pediatric offices delivering an office specific access code. This procedure ensures acknowledgment of the treating pediatrician.

The access code of each office gives the parent the possibility to generate a random but specific family code. This family code gives the opportunity to share access to further family members, so that these people could view and enter in the same profiles. Even though it is a random pseudonym, it could nevertheless be identifying if it is made available to others by the owner. If it is shared with the treating pediatric office, it enables comparison with reference records in selected participating offices.

### 2.3. Study Design

A challenge of EMA is that there is no other reliable source data, since the EMA is considered the source data. Therefore, the question arises which further data sources could be an alternative to the source data for evaluation of the data quality as it is know from classical registries.

Two possible data sources emerge for the FeverApp registry: data from a physician’s office and additional retrospective reports with data providers. Both approaches to data quality assurance were implemented. First, data matching with one practice was conducted [15]. As a second quality assurance concerning the completeness of the observation units children and fever phases as well as the recording of medication, we conducted a voluntary survey among completing parents to check the reliability of these essential variables by two response time points (momentary and retrospective). For both sources, none of these additional sources can be considered as valid as the source data of a clinical trial, since the EMA data are basically the actual source data of the registry. To ensure the equivalence of the ecological momentary assessment, the participating parents were asked to reassess their documentation in a separate survey. Of course, the parents contacted were also able to review their entered data at the time of completing the questionnaire. The questionnaire and the data can be found in the supplementary materials.

In this observational study, we tested the reliability of parental entries in the FeverApp during the year 2020. Parents with known email address were invited on 23 January 2021 to report the fever events in 2020 and their anticipated usage of the app in 2021. In January 2022 the real usage of the FeverApp in 2021 was compared with the anticipated usage.

Only FeverApp users from the first participating office with informed consent or those who had voluntarily provided their email addresses since October 2020 were included. The first medical office started the distribution already in September 2019. Intrinsically motivated persons tend to persist longer on tasks which yields better performance of assignment according Cerasoli et al. [16].Therefore, the app users with voluntarily provided email addresses show their interest and willingness for contact with the researchers, and the answers provided by them.

The survey contains two closed introductory questions to collect information on the number of children and inquiries about any arbitrary test entries made. The number of children in the family is taken into account so that the questionnaire is individualized for each participant. The main part contains one obligatory question concerning submitted fever episodes and three additional questions concerning medications if any fever episodes were entered and two open questions for explanation why not all fever episodes or medications have been entered if participant stated that not all fever events or medications have been submitted. These up to four items could be answered multiple times if multiple children are observed. The exact number of fever events is not asked. Three further items cover whether the FeverApp adds benefit for parents, whether parents would continue to use the app in 2021, and a possibility to give verbal feedback. The survey questions can be found in the supplement.

The data analysis is performed using the statistical software R [17] and follows the questions in the survey. Demographic and genuineness data are presented as descriptive statistics. Free text entries were treated as hints and categorized into four categories: negative, positive, neutral and suggestion with the R-package *sentiws* and following manual review.

Reliability is determined for both observation units, i.e., amount of children and amount of fever episode, as well as medication. Cohen’s Kappa (κ) [18] is a statistical measure to calculate intra-rater reliability. It can be interpreted according to Greve and Wentura [19], where κ ≥ 0.75 indicates good to excellent agreement, similar to Landis and Koch [20] who suggested the following interpretation of κ: <0.20 = “poor”, 0.21–0.40 = “fair”, 0.41–0.60 = “moderate”, 0.61–0.80 = “substantial”.

We assume the information concerning number of children given in the survey as more reliable than the number of registered children in the FeverApp because it is not obligatory for users to register all their children. Therefore, we count the number of children in the survey as reference. Otherwise, regarding the definition of sensitivity and specifity, the presence of submitted information about fever events, medication and further usage in the FeverApp data is more reliable than assertions of the survey participants.

Attitudes toward anticipated usage and sentiment analysis of free text messages regarding the FeverApp are presented at the end.

### 2.4. Ethical Consideration

Ethical approval by an independent ethics committee of the University of Witten/Herdecke on pseudonymized data collection using an app has been received (#139/2018), as well as a positive vote by the data protection service.

## 3. Results

### 3.1. Characteristics of Participants

**Figure 2:**
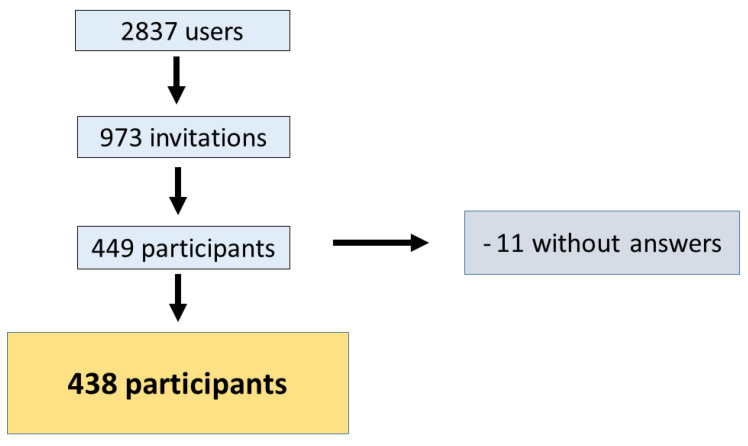
Data cleaning.

The response rate amounts to 45%: 438 of 973 participants (Figure 2) registered in the FeverApp with email addresses participated in the survey (they make up 34.3% of 2837 families registered at the time). Children of 71.3% (312) survey participants are patients from a large pediatric office, which is participating since autumn 2019.

**Figure 3:**
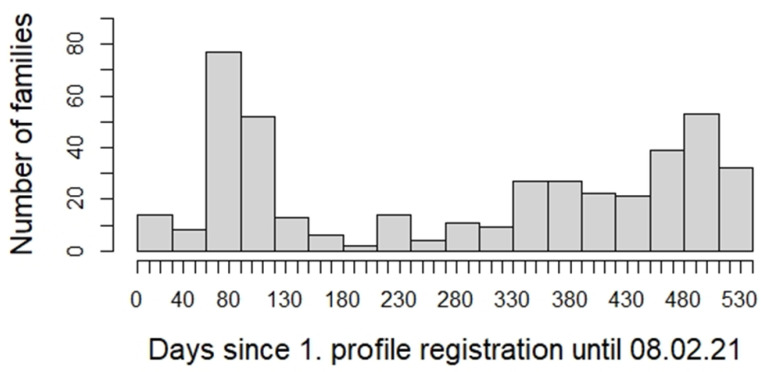
Participation time since first profile registration in days.

The remaining 28.7% (126 participants) origins from 52 pediatric offices that joined the FeverApp project a year later, in 2020, so that the histogram of duration since first profile registration in days is bimodal (Figure 3). According to the registered profiles, 50% of the participants used the app for a maximum of one year and 25% of the participants used the app only three months or even less.

In all participating families, only one person responded to this voluntary survey. However, in 25 families both parents are registered. It is not possible to detect in such cases which member of the family submitted the answers in the survey if nobody in the family has submitted any fever episodes. We are only able to characterize the sample including all users from each of 438 families using information provided in the FeverApp registry. Overall, these are 463 users.

The interquartile range (IQR) of age in the sample is between 32 and 39 years with 35 as median age. The set of all users (N *=* 2679) of the FeverApp to the time point 31 January 2021 has an IQR between 31 and 38, with median age of 34. The Mann–Whitney U-Test shows that the difference is significant: (*p*-value < 0.001), however this difference is not relevant.

In the 438 participating families, mothers form the majority. The distribution of roles in the families from the sample does not differ from the set of all users according to the Fisher’s exact test (*p*-value = 1).

About 98% of users from the sample of families have answered the question about the school graduation (Table 1): the majority of users have a high school diploma with qualification for university admission. These results are common with the set of all users to the time point of 31 January 2021 (Fisher’s exact test with *p*-value = 1).

### 3.2. Comparison of Answered and Registered Number of Children

The number of children in the family was the first question in the survey. Twelve participants (2.7%) ignored the question. The 208 of 426 participants (48.8%) only have one child, 169 participants raise two children (39.7%). Multi-child families account for just over 10% of the total: 41 participants with three children (9.6%), seven participants with four children (1.6%) and one family with five children (0.2%). Overall, these are 717 children in the participants’ families.

In this FeverApp sample are 649 profiles registered by the participating parents (with four synonym profiles and seven adults). Completeness of observation units is 89%.

Table 2 describes the agreement between the answers of the participants in the survey with the number of registered children in the FeverApp. Cohen’s κ is 0.74 (substantial agreement) with confidence interval [0.69; 0.80]. If no entry or answer were made, we define this as 0 children.

The comparison of the two sources shows that 363 participants (82.9%) have registered all their children: there is no difference between answers in the app and the survey. 56 (12.7%) participants have not registered all their children: the difference is positive; they state to have more children than they have registered in the app. Only seven participants state to have less children than profiles: in three cases, the registered profiles belong to the parents themselves, and in four cases to other children (e.g., children of friends). The absolute numbers are summarized in Table 3.

### 3.3. Gender and Age of Children According to the App

The sample of registered children in the FeverApp consists of 328 boys and 310 girls. The age of the 638 children according to the answers in the FeverApp is between 2.3 months and 17 years. The median age is 44.3 months (3.7 years) with an IQR from 19.3 (1.6 years) to 73.3 months (6.1 years). The Mann–Whitney U-test (*p*-value < 0.01) shows that the app is used especially by participants with young children with a median age of 3.2 years, compared to participants without any entries in the app where the median age of children is 4.4 years.

### 3.4. Genuineness of Entries According to the Survey Compared with the App

Of the 438 participants, 325 (74.2%) stated that they make only genuine entries in the app. The number of participants who do not answer the question about genuineness of the entries in the app was 58 (13.2%). All others stated that they make either only test entries or only some genuine entries. In fact, most participants who did not answer the question (50 of 58) or stated to do only test entries (27 of 34), have no entries in the app. On the contrary, many participants who stated that they make only genuine entries or only some true entries have at least one entry in the app (Table 4).

About three quarters stated to perform exclusively genuine entries in the app and 65.5% (287 of 438) of these actually have entries already in the app. This majority may be increased with further 13.2% without response on this question, probably due to the short observation period in some participants without any possible entries. Of these 7.8% with exclusive test entries, most performed no entries (79.4%). Hence, the number would increase if only families with existing entries in the app were counted as denominators.

### 3.5. Comparison for Fever Episodes and Medication

Of the 438 participants, 300 report fever episodes and 95 do not (90.0% agreement between survey and app, Cohen’s κ *=* 0.75 (substantial agreement) with confidence interval [0.66; 0.82]) (Table 5). Specificity is 80.5% and sensitivity 93.8%.

Whereas medication has 73.7% agreement and κ *=* 0.49 (moderate agreement [16,17]) with confidence interval [0.42; 0.54], 137 participants report medication and 186 do not (Table 6). Specificity is 65.0% and sensitivity 90.1%.

### 3.6. Benefit of the App and Usage in 2021

Only 9.4% of the participants do not consider the app as a personal benefit, 87.3% would like to continue using the app in 2021. It is to be noted that 112 (90.3%) of 124 users, who do not know whether the app had a benefit for them, would like to continue to use the app in 2021 (Table 7).

### 3.7. Evaluation of Free Text Response

One fifth of the 438 respondents (n = 97, 22.1%) made free text entries. The majority gave positive feedback (n = 56, 57.7%) like “The app means for me a security in dealing with fever.” or neutral feedback (19, 19.6%) feedback e.g., “I will enter fever incidents into the app” as well as concrete suggestions (n = 9, 9.3%) like “It would be nice if the doctor had access to the app.” Only 13 (13.4%) of the 438 respondents (3.0%) gave negative feedback e.g., “The entries are laborious and time-consuming”. The users with negative feedback concern about technical issues and time-consuming process of submission especially of the delivered medication. The users with neutral messages had no fever episode or feel confident in fever management without the app. Suggestions were made by users who like the app in general but would like to improve performance or to launch additional functions. Users with positive feedback praise the app for convenient use, provision of self-confidence in fever management and the possibility to use the app as a fever diary.

## 4. Discussion

This study aims to assess reliability of EMA in an app-based registry by an email-based survey for the observation units of children and fever episodes as well as the recording of medication. Additionally, a descriptive analysis of the users’ attitudes toward the FeverApp and their sociodemographic features and sentiment analysis of the text messages was performed. The comparison of the sample with the population of all families using the app has shown that the sample is a good representation of the whole set of the FeverApp users [7].

The agreement for registered children is substantial because it is not obligatory to register all children in the family, however desirable by the researchers. The agreement for submitted fever episodes is also substantial. This confirms the reliability of this data capturing method. It is a much higher effort to enter medication, which is also confirmed via text messages of some respondents; this may explain the moderate agreement of submitted medicaments. Regarding the existence of documentation for medication, a substantial agreement of 73.7% was found, due to the reason that documentation of medication is not seen as mandatory.

We observe interesting features of the users. The majority of survey participants had only one child or two children. This explains the high agreement. Especially participants with young children under three years of age use the FeverApp, so that 75% of the survey participants` children are 6 years old or younger. It can be concluded, that parents of infants and toddlers are the most relevant target group. Parents with additional older children may not register them, due to lack of fever in this age group. This is supported by the age analysis that was performed above. The FeverApp project addresses in the first line families with young children because feverish illnesses are common especially in early childhood [21,22].

The participants who refused to answer the question about genuine entries mostly did not give any entries in the app what explains their behavior.

Interestingly, most of the users, who did not conclude whether the app had any benefit for them, would like to continue to use the app in 2021. This underlines the general interest of users, even though they could not assess the multi-functionality of the app.

There are not many studies which evaluate reliability of app-based data. Studies of Fortea et al. [23] and Hyun et al. [24] are dedicated to validity and reliability of EMA data. Data quality of EMA were already analyzed by Wu et al. [25] where they evaluate test-retest-reliability of the EMA in audiology research. Welling et al. [26] focus on the detection of careless responses in EMA with post-hoc analysis. The meta-analysis study by Cerino et al. [27] is dedicated to response bias in EMA studies.

### Limitations

Several limitations of the study must be considered. Regarding arbitrary test entry, we rely completely on the information of users and cannot validate the answer additionally. Similarly, the benefit assessment is subjective. It is not possible to verify if participants make entries for all fever episodes and medicaments or only for some of them. This may be due to lack of time or forgetfulness.

The sample of participants may be biased, because it needs to rely on an existing and acknowledged email address. For a part (28.7%) of the participants, the observation period was short. This is due to the reason, that the option to report an email as contact possibility was implemented in the FeverApp four months before the survey was conducted. The families take advantage of the offer to use the app voluntarily and only if they are interested in the FeverApp, so the number of the respondents could be higher if the submission of the email address was possible earlier.

Moreover, due to the COVID-19 pandemics the opportunity to make entries has become rare: the reduction of contacts or even complete social isolation of children has resulted in a decrease of diseases typically accompanied with fever [13,28]. Increased number of contacts after COVID-19 pandemic is associated with rebound of feverish illnesses after COVID-19 and poses a challenge for pediatricians [29].

## 5. Conclusions

Our study evaluates test-retest reliability with an email-based survey. A similar study was performed by Goueth et al. [30]. The results show that the reliability of data from the FeverApp registry is comparable with the clinical data collected in practice [31,32] and research [33,34].

Email surveys are a possible approach to evaluate EMA based registry data. Data concerning fever episodes show an adequate reliability in the FeverApp registry. In the future, we plan to repeat the survey under common circumstances and to analyze the factors influencing the agreement. With this approach, surveys of further samples and variables could help to improve the quality of this EMA based registry.

## Figures and Tables

**Table 1:** Sociodemographic parameters of survey participants and all FeverApp users.

	Survey Participants			All FeverApp Users		
		n	Missing Information		N	Missing Information
Age in years (median, IQR)	35 (32–39)	463	0	34 (31–38)	2542	137
Role in the family (%, n)		462	1		2553	126
Mother	88.1 (407)			81.4 (2077)		
Father	11.3 (52)			17.4 (444)		
Grandmother	0			0.1 (3)		
Grandfather	0			0.2 (6)		
Other	0.6 (3)			0.9 (23)		
School degree (%, n)		454	9		2506	173
Qualification for university admission	57.7 (262)			50.0 (1254)		
Advanced technical college entrance qualification	22.3 (101)			20.9 (525)		
General certificate of secondary education	17.6 (80)			22.0 (552)		
Secondary modern school	2 (9)			6.1 (154)		
No regular school-leaving certificate	0.4 (2)			0.8 (21)		

**Table 2:** Contingency table for the number of children in families.

		Children According Survey						
		0	1	2	3	4	5	Total
**Children according FeverApp**	**0**	0	2	3	2	0	0	7
	**1**	8	203	28	8	4	0	251
	**2**	3	2	134	8	0	0	147
	**3**	0	1	4	23	0	0	28
	**4**	1	0	0	0	3	1	5
	**5**	0	0	0	0	0	0	0
	**Total**	12	208	169	41	7	1	N = 438

**Table 3:** Differences between the number of registered children per family in the app and reported in the survey.

Difference	−2	−1	0	1	2	3	No Report in the Survey
**Counts**	1	6	363	39	11	6	12
	More in the app				More in the survey		

**Table 4:** Reported genuineness of entries.

Genuineness	Own Intention in Survey		Real Recorded Entries in the App	
Do you Make Test Entries?	Absolute Frequency	Relative Frequency of Total in %	Absolute Frequency	Relative Frequency of Intention in %
**No: Only genuine answers**	325	74.2%	287	88.3%
**Yes: Occasional test entries**	21	4.8%	18	85.7%
**Yes: Only test entries**	34	7.8%	7	20.6%
**Missing response**	58	13.2%	8	13.8%
Total	438	100%	320	73.1%

**Table 5:** Reported fever episodes.

Fever Entries	App: Participants with Entries	App: Participants without Entries	Total
**Survey: Participants with entries**	300 (true positive)	23 (false positive)	323
**Survey: Participants without entries**	7 (false negative) + 13 (without answer)	89 (true negative) + 6 (without answer)	115
Total	320	118	438

**Table 6:** Reported medication delivery.

Medication	App: Participants with Entries	App: Participants without Entries	Total
**Survey: participants with entries**	137 (true positive)	100 (false positive)	237
**Survey: Participants without entries**	6 (false negative) + 9 (without answer)	66 (true negative) + 120 (without answer)	201
Total	152	286	438

**Table 7:** Attitudes toward usage of the app in 2021 and additional benefits of the app.

Benefit/Usage in 2021	No Answer	Yes, I Will Use It	No, I Will Not Use It	I Do Not Know	Total
**No answer**	16	10	0	2	28 (6.4%)
**Yes, the app has additional benefit for me**	1	240	2	2	245 (55.9%)
**The app has no additional benefit for me**	0	21	9	11	41 (9.4%)
**I do not know**	0	112	0	12	124 (28.3%)
Total	17 (4.0%)	383 (87.3%)	11 (2.5%)	27 (6.2%)	438

## Data Availability

The data presented in this study are available in SPSS format as Supplementary Material.

## Supplementary Materials

The following supporting information can be downloaded at: https://www.mdpi.com/article/10.3390/children10020385/s1, translated survey questionnaire; data set in SPSS format.
