# Supplementary material for: Reliability of Data Collected via Ecological Momentary Assessment on the Example of FeverApp Registry
Source: Children (Basel). 2023 Feb 15;10(2):385. doi: 10.3390/children10020385 (PMC9954832; doi:10.3390/children10020385)
Supplement: Supplementary file 1 [file children-10-00385-s001.zip › Survey_Questionnaire.pdf]

**Questionnaire:**

**Intro**

1. How many children do you have? (under 18 years). Please select only one of the following answers:
    - 1
    - 2
    - 3
    - 4
    - 5
2. Have you made any test entries when using the FeverApp? Please select only one of the following answers:
    - I have made only test entries.
    - I have made test entries and genuine entries.
    - I have made only genuine entries.

**Fever events**

3. Have you entered all fever events for your 1st child since the app was activated? Please select only one of the following answers:
    - Yes, all fever events have been entered.
    - There were no fever events.
    - No, not all fever events were entered.
    - No profile has been created for the child.
4. Why were not all fever events entered? Answer this question only if the following conditions are met: Answer was 'No, not all fever events were entered' for question 3. “Have you entered all fever events for your 1st child since activating the app?” Please enter your answer here:

*Answer this question only if the following conditions are met: Answer was 'Yes, all fever events have been entered' or 'No, not all fever events entered' for question “Have you entered all fever events for your 1st child since the app was activated? “*

5. Have you entered all medications given in the fever context for your 1st child? Please select only one of the following answers:
    - Yes, all medications have been entered.
    - No medication has been administered.
    - No, not all medications have been entered.

6. Why were not all medications entered? Answer this question only if the following conditions are met: Answer was 'No, not all medications were entered' for question 5 “Did you enter all medications administered in the fever context for your 1st child?” Please enter your answer here:

*Answer the following question only if the following conditions are met: Answer was equal to or greater than 2 for question “How many children do you have? (under the age of 18)*

7. Have you entered all fever events for your 2d child since the app was activated? Please select only one of the following answers:
    - Yes, all fever events have been entered.
    - There were no fever events.
    - No, not all fever events were entered.
    - No profile has been created for the child.

8. Why were not all fever events entered? Answer this question only if the following conditions are met: Answer was 'No, not all fever events were entered' for question 7 “Have you entered all fever events for your 2d child since activating the app?” Please enter your answer here:

*Answer the following question only if the following conditions are met: Answer was 'Yes, all fever events have been entered' or 'No, not all fever events entered' for question 7 “Have you entered all fever events for your 2d child since the app was activated? “*

9. Have you entered all medications given in the fever context for your 2d child? Please select only one of the following answers:
    - Yes, all medications have been entered.
    - No medication has been administered.
    - No, not all medications have been entered.

10. Why were not all medications entered? Answer this question only if the following conditions are met: Answer was 'No, not all medications were entered' for question 9 “Did you enter all medications administered in the fever context for your 2d child?” Please enter your answer here:

*Answer the following question only if the following conditions are met: Answer was equal to or greater than 3 for question “How many children do you have? (under the age of 18).*

11. Have you entered all fever events for your 3d child since the app was activated? Please select only one of the following answers:
    - Yes, all fever events have been entered.
    - There were no fever events.

- No, not all fever events were entered.
- No profile has been created for the child.

12. Why were not all fever events entered? Answer this question only if the following conditions are met: Answer was 'No, not all fever events were entered' for question 11 “Have you entered all fever events for your 3d child since activating the app?” Please enter your answer here:

*Answer the following question only if the following conditions are met: Answer was 'Yes, all fever events have been entered' or 'No, not all fever events entered' for question 11 “Have you entered all fever events for your 3d child since the app was activated? “*

13. Have you entered all medications given in the fever context for your 3d child? Please select only one of the following answers:
    - Yes, all medications have been entered.
    - No medication has been administered.
    - No, not all medications have been entered.

14. Why were not all medications entered? Answer this question only if the following conditions are met: Answer was 'No, not all medications were entered' for question 13 “Did you enter all medications administered in the fever context for your 3d child?” Please enter your answer here:

*Answer the following question only if the following conditions are met: Answer was equal to or greater than 4 for question “How many children do you have? (under the age of 18).*

15. Have you entered all fever events for your 4th child since the app was activated? Please select only one of the following answers:
    - Yes, all fever events have been entered.
    - There were no fever events.
    - No, not all fever events were entered.
    - No profile has been created for the child.

16. Why were not all fever events entered? Answer this question only if the following conditions are met: Answer was 'No, not all fever events were entered' for question 15 “Have you entered all fever events for your 4th child since activating the app?” Please enter your answer here:

*Answer the following question only if the following conditions are met: Answer was 'Yes, all fever events have been entered' or 'No, not all fever events entered' for question 15 “Have you entered all fever events for your 4th child since the app was activated? “*

17. Have you entered all medications given in the fever context for your 4th child? Please select only one of the following answers:
    - Yes, all medications have been entered.
    - No medication has been administered.
    - No, not all medications have been entered.

18. Why were not all medications entered? Answer this question only if the following conditions are met: Answer was 'No, not all medications were entered' for question 17 “Did you enter all medications administered in the fever context for your 4th child?” Please enter your answer here:

*Answer the following question only if the following conditions are met: Answer was equal 5 for question “How many children do you have? (under the age of 18).*

19. Have you entered all fever events for your 5th child since the app was activated? Please select only one of the following answers:
    - Yes, all fever events have been entered.
    - There were no fever events.
    - No, not all fever events were entered.
    - No profile has been created for the child.

20. Why were not all fever events entered? Answer this question only if the following conditions are met: Answer was 'No, not all fever events were entered' for question 19 “Have you entered all fever events for your 5th child since activating the app?” Please enter your answer here:

*Answer the following question only if the following conditions are met: Answer was 'Yes, all fever events have been entered' or 'No, not all fever events entered' for question 19 “Have you entered all fever events for your 5th child since the app was activated? “*

21. Have you entered all medications given in the fever context for your 5th child? Please select only one of the following answers:
    - Yes, all medications have been entered.
    - No medication has been administered.
    - No, not all medications have been entered.

22. Why were not all medications entered? Answer this question only if the following conditions are met: Answer was 'No, not all medications were entered' for question 21 “Did you enter all medications administered in the fever context for your 5th child?” Please enter your answer here:

**Outro:**

23. Does the FeverApp add benefit for you? Please choose only one of the following answers:
    - Yes
    - No
    - I do not know

24. Will you continue to use the FeverApp in 2021? Please choose only one of the following answers:
    - Yes

- No
- I do not know

25. Is there anything else you would like to tell us? Please enter your answer here:
